# The effects of notch filtering on electrically evoked myoelectric signals and associated motor unit index estimates

**DOI:** 10.1186/1743-0003-8-64

**Published:** 2011-11-23

**Authors:** Xiaoyan Li, William Z Rymer, Guanglin Li, Ping Zhou

**Affiliations:** 1 Sensory Motor Performance Program, Rehabilitation Institute of Chicago, Chicago, USA; 2 Department of Physical Medicine and Rehabilitation, Northwestern University, Chicago, USA; 3 Research Center for Neural Engineering, Institute of Biomedical and Health Engineering, Shenzhen Institutes of Advanced Technology, Chinese Academy of Sciences, Shenzhen, China

## Abstract

**Background:**

Notch filtering is the most commonly used technique for suppression of power line and harmonic interference that often contaminate surface electromyogram (EMG) signals. Notch filters are routinely included in EMG recording instrumentation, and are used very often during clinical recording sessions. The objective of this study was to quantitatively assess the effects of notch filtering on electrically evoked myoelectric signals and on the related motor unit index measurements.

**Methods:**

The study was primarily based on an experimental comparison of M wave recordings and index estimates of motor unit number and size, with the notch filter function of the EMG machine (Sierra Wave EMG system, Cadwell Lab Inc, Kennewick, WA, USA) turned on and off, respectively. The comparison was implemented in the first dorsal interosseous (FDI) muscle from the dominant hand of 15 neurologically intact subjects and bilaterally in 15 hemiparetic stroke subjects.

**Results:**

On average, for intact subjects, the maximum M wave amplitude and the motor unit number index (MUNIX) estimate were reduced by approximately 22% and 18%, respectively, with application of the built-in notch filter function in the EMG machine. This trend held true when examining the paretic and contralateral muscles of the stroke subjects. With the notch filter on vs. off, across stroke subjects, we observed a significant decrease in both maximum M wave amplitude and MUNIX values in the paretic muscles, as compared with the contralateral muscles. However, similar reduction ratios were obtained for both maximum M wave amplitude and MUNIX estimate. Across muscles of both intact and stroke subjects, it was observed that notch filtering does not have significant effects on motor unit size index (MUSIX) estimate. No significant difference was found in MUSIX values between the paretic and contralateral muscles of the stroke subjects.

**Conclusions:**

The notch filter function built in the EMG machine may significantly reduce the M wave amplitude and the MUNIX measurement. However, the notch filtering does not jeopardize the evaluation of the reduction ratio in maximum M wave amplitude and MUNIX estimate of the paretic muscles of stroke subjects when compared with the contralateral muscles.

## Introduction

Surface electromyogram (EMG) recordings are used for assessing overall muscle activity in various disease states. The noninvasive nature and easy-to-use features of the surface recording technique contribute to its widespread application in various fields such as biofeedback, movement analysis, physical rehabilitation, ergonomics, occupational and sports medicine [1]. The value of surface EMG recording for the quantification of both voluntary and electrically elicited contractions has been demonstrated by many investigators.

It is not uncommon that during the recording process, the quality of EMG signals is compromised by interfering noise originating from the power line and other sources. The subsequent distortion of the surface EMG signal and the removal of the power line and other interference have received considerable attention [2-7]. Different methods have been developed for power line and harmonic noise suppression including the most commonly used multiple notch filters centered on the power line and harmonic frequencies [5,7]. Other forms of time domain and frequency domain filters (e.g., a matched filter and a frequency domain Hampel filter) have also been implemented for this purpose [2,4]. Since the frequency of the interfering signal falls within the bandwidth of the surface EMG signal, adaptive filtering has also been developed to reject the unwanted noise while leaving the surface EMG signal relatively intact [7,8].

It is worth noting that virtually all previous EMG studies that focused on assessing and suppressing power line and harmonic noise targeted voluntary surface EMG signals, while little attention has been given towards electrically elicited signals. Electrically evoked EMG or M wave recordings have many important applications in both neurophysiological research and clinical electrodiagnosis. For example, the ratio of the maximum peak-peak amplitude of the H-reflex to the M wave can be considered as an index of excitability of the H-reflex arc [9,10]. Due to the deterministic nature and the small variance of the signal, M wave recording is also considered as a potentially preferable approach to voluntary surface EMG methods for assessing muscle fatigability [11,12]. Visual inspection and computer aided quantification of morphological features of the M wave can also be used to explore the physiological properties of a muscle and their alterations in pathological states [13-15]. M wave recording is also a critical source of information regarding potential motoneuron loss and for tracking motoneuron disease progression. It forms the basis of various motor unit number estimation (MUNE) techniques [16,17], or for measures using the recently developed index techniques that solely require several maximum electrical stimulations [18-20].

The methodologies described above are based on the assumption that it is possible to make reliable measurements of the M wave. The artifacts in the voluntary surface EMG signals also routine exist in the electrically evoked myoelectric signals. The electrical stimulation may impose extra artifacts in the recorded EMG signal. Moreover, M wave or compound muscle action potential (CMAP) is often used as a diagnostic tool in a clinical environment, where electrical power supplies are prevalent. Thus, the surface EMG electrode may inevitably pick up electromagnetic noise [3]. In such a situation, suppression of power line and harmonic interference is required to have uncontaminated M wave recordings. In fact, most of the clinical EMG machines have a built-in-notch filtering function, optional to operators. Given the above, there are surprisingly no studies to our knowledge that have investigated the effects of imposing such a noise reduction processing on the M wave and other related measures and calculations. Most of the previous studies have focused on simple test-retest reliability, including two studies performing comprehensive analysis of M wave reliability using the intraclass correlation coefficients [11,15,21]. During our previous studies [22], we noted that the maximum M wave amplitude of our subjects tended to be low compared with the values reported by others [23,24], potentially due to the application of the system notch filtering function in the EMG machine. However, the quantitative analyses of the effects of notch filtering on M wave and other related measurements are lacking.

In light of this deficiency, the purpose of our study was to examine how the most commonly used notch filter for power line interference suppression could influence M wave recordings. The amplitude, or the area of the negative phase of the M wave, plays a critical part in estimating the motor unit numbers in a muscle [16,17]. We thus chose to examine the influence of notch filtering on these parameters. We also explored how the notch filter could change the motor unit number index (MUNIX) estimate, a recently developed neurophysiological technique that relies on maximum M wave and voluntary surface EMG signals for computing an index proportional to the number of motor units in a muscle [19,20]. Finally, to investigate the effects of notch filtering on assessment of muscle fiber or motor unit loss, we compared the findings in the presence and absence of the notch filter functions when using M wave and MUNIX measurements to examine the paretic and contralateral muscles of stroke survivors.

## Methods

### A. Subjects

Fifteen neurologically intact subjects (9 males, 6 females, 41.5 ± 13.7 years) and 15 subjects (8 males, 7 females, 59.2 ± 11.2 years) who sustained hemiparetic stroke participated in this study. All our stroke subjects were recruited from the Clinical Neuroscience Research Registry at the Rehabilitation Institute of Chicago (Chicago, IL, USA). A screening examination and clinical assessment were performed by a physical therapist to determine the eligibility for each stroke subject. Inclusion criteria for participation of the study include age between 21-75 years old; experience of stroke with initial onset more than 6 month; medically stable with clearance to participate; ability to provide informed consent, with Mini-Mental State Examination (MMSE) must be 23 or higher. Exclusion criteria include history of spinal cord injury or traumatic brain damage; inability to comprehend conversations; history of serious medical illness such as cardiovascular or pulmonary complications; history of severe motion sickness; and any condition that, in the judgment of a physician, would prevent the person from participating. Women who are pregnant or nursing were excluded from the study. Among the 15 stroke subjects, the left limb was affected in 7 subjects and the right limb was affected in 8 subjects. The duration between the stroke onset and the experiment time was 11.7 ± 7.5 years (range: from 10 months to 24 years and 6 months). The 15 stroke subjects showed a Chedoke score of 3 ± 1, and a Fugl-Meyer (hand) score of 7 ± 5. The study was approved by the Institutional Review Board of Northwestern University (Chicago, IL, USA). All subjects gave their written consent before the experiment.

### B. Experiments

Experiments were performed on the first dorsal interosseous (FDI) muscle of the dominant hand of the neurologically intact subjects, and bilaterally in all the hemiparetic stroke subjects. Subjects were seated comfortably in a chair with the examined forearm placed in its natural, resting position on a height-adjustable table. They were instructed to relax at the wrist, elbow and shoulder. The hand and forearm were held in a vertical half supinated position. Hand skin temperature was not specifically monitored during the experiment. A thermometer showed a constant temperature (approximately 72 degrees Fahrenheit) in the laboratory.

Prior to the recording, the skin surfaces over the ulnar aspect of the wrist, the back of the hand, and the index finger were lightly abraded and cleaned with rubbing alcohol to facilitate the recording. A small amount of conductive electrode cream was used to reduce skin-electrode impedance. Care was taken not to leave any on the skin to avoid short-circuiting the electrodes.

The maximum M wave or CMAP was recorded first. Evoking the maximum M wave by supramaximal stimulation is the electrical equivalent of recruiting of all motor units within a muscle innervated by the stimulated nerve. A maximum M wave from the FDI muscle was obtained by stimulation of the ulnar nerve at the wrist, using an intensity sufficient to elicit a maximum CMAP. The primary equipment used for this recording was the Sierra Wave EMG system (Cadwell Lab Inc, Kennewick, WA, USA). A remote handheld stimulator with a StimTroller was used to generate stimuli through a cathode (a 10 mm silver/silver chloride pole).

Two 10 mm silver/silver chloride disc surface recording electrodes were used to record electrical activity from the FDI muscles. Electrode placement was similar to that for standard ulnar motor studies. The active surface electrode was positioned over the motor point of the FDI muscle with the reference surface electrode positioned over the second metacarpophalangeal (MCP) joint. An adhesive ground electrode was placed on the back of the hand. All the surface electrode positions were further reinforced with surgical tape to reduce electrode movement during the recording.

The ulnar nerve was stimulated about 2 cm proximal to the wrist crease. The duration of each stimulus was 200 μs. Different from the stimulus protocol used for traditional MUNE methods (where the stimulus intensity usually starts below the response threshold and increases in very small increments until the maximum M wave is achieved), in our MUNIX study the stimulation intensity started around 15-20 mA. The intensity was further increased in increments of approximately 20% above that until the stimulation intensity eliciting the maximal response was reached. Then, the stimulation intensity was increased to 120% of the final intensity to confirm that no further increase in the peak-to-peak amplitude of the M wave. Such a use of approximately 20 percent supramaximal stimulation intensity guarantees the activation of all the motor axons innervating the muscle. Previous studies demonstrated low CMAP amplitudes from suboptimal electrode placement (or nerve stimulation) may yield erroneously low MUNIX values [18]. Therefore, to ensure that the CMAP amplitude is maximized throughout the MUNIX study, during the experiment, the electrode placement was optimized by testing several different locations. In addition, re-cleaning of the skin and reapplication of the electrode cream were performed as necessary (to guarantee the best recording quality).

With all the electrodes maintained at the same position, after the maximum M wave recording, voluntary surface EMG signals were recorded from the FDI muscle while the subject generated an isometric muscle contraction force at 5-10 different levels (representing minimal to maximal effort). The force levels were defined qualitatively by the examiner, offering resistance in abduction to the contracting FDI muscle. The different force levels were recorded using a single trial with graded contractions consisting of the required EMG epochs distributed from minimal to maximal effort. Subjects were allowed substantial rest to avoid muscle fatigue during the recording.

For all subjects, the M waves and voluntary surface EMG responses were sampled at 2 KHz. To investigate the effects of notch filtering on M wave recording and other related calculations, the maximum M wave was recorded with the built-in-notch filter (1^st ^order filter, rejected frequency 60 Hz) function of the EMG machine on, and repeated with the notch filter off. The notch filter was turned off for voluntary surface EMG recordings. Responses recorded by the electrodes were amplified by a differential AC amplifier. A split screen sensitivity was set at 2 mV/division in the M wave zone. Sweep speed was 5 ms/division. All signals were recorded to a hard disk and analyzed offline.

### C. Data Analysis

The maximum M wave and different levels of voluntary surface interference pattern (SIP) EMG were used to compute the MUNIX for the examined FDI muscle [19,20]. The area and power of the maximum M wave were first computed. Then, the voluntary surface EMG signals were examined, and those SIPs with high frequency noise, power line interference, baseline shift or other artifacts were excluded from the analysis. The remaining SIP signals were used to calculate the average area and power of the SIP for a one-second epoch. This analysis was performed for each voluntary contraction level. The values calculated from the maximum M wave and different levels of SIPs were used to compute the "ideal case motor unit count (ICMUC)":

(1) $$ICMUC=\frac{Maximum\;M\;Wave\;Power\times SIP\;Area}{Maximum\;M\;Wave\;Area\times SIP\;Power}$$

Thus, each level of SIP gave two results: SIP area and ICMUC. Regression analysis was then used to define the relationship between SIP area and ICMUC by the following formula:

(2) $$ICMUC=\beta {\left(SIP\;Area\right)}^{\alpha }$$

The parameters β and α obtained from the regression were used to compute the MUNIX [19,20]:

(3) $$MUNIX=\beta {\left(20\right)}^{\alpha }$$

In MUNIX analysis, it should be noted that very low amplitude voluntary surface EMG signals can give very high ICMUC values. To exclude this artifact, three criteria were imposed to accept an SIP epoch [18]: (1) SIP area > 20 mVms; (2) ICMUC < 100; and (3) SIP area/CMAP area > 1.

With MUNIX values available, the motor unit size index (MUSIX) could be obtained by dividing MUNIX into the maximum M wave amplitude [18]:

(4) $$MUSIX=\frac{Maximum\;M\;wave\;amplitude}{MUNIX}$$

MUSIX, measured in volts, is an index that reflects the average amplitude of the individual surface motor unit action potentials (MUAPs).

We measured the maximum M wave amplitude, the MUNIX and MUSIX values in the dominant FDI muscles of neurologically intact subjects and bilaterally in hemiparetic stroke subjects, with the notch filtering function turned on and off for M wave recordings respectively. We determined whether the notch filtering function has significant effects on M wave recording and motor unit index measurement. We specifically examined how such a filtering function may affect our evaluation of muscle fiber or motor unit loss in paretic muscles by comparing the measured parameters with the contralateral muscles, in the presence and absence of the notch filtering function. The analysis of variance (ANOVA) was used for statistical analysis. The significance level was defined as p < 0.05.

## Results

### Results from neurologically intact subjects

**Figure 1:**
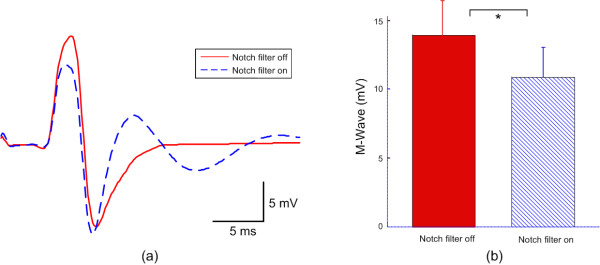
**The effects of the notch filtering function on M wave recording from FDI muscles**. (a) A comparison of typical M waves with and without notch filtering; (b) Bar plot of M wave amplitude across all subjects with and without notch filtering. "*" indicates significant difference (p < 0.001).

Recording of maximum M waves and voluntary surface EMG signals at different levels of contraction were obtained from dominant hand FDI muscles of all the intact subjects with or without the notch filtering function turned on. For all the intact subjects, we observed a significant decrease in maximum M wave amplitude when notch filter was on, as compared with observations made with the filter off (Figure 1). As Figure 1a illustrates, in addition to reduced amplitude and area of the first negative phase of the M wave, the M wave shape tends to change from two major phases to multiple phases. Across all intact subjects (Figure 1b), the maximum M wave amplitude of the FDI muscle was 10.8 ± 2.1 mV (range: 6.2-13.8 mV) for notch filtering on and 13.9 ± 2.4 mV (range: 8.4-16.7 mV) for notch filtering off (p < 0.001).

**Figure 2:**
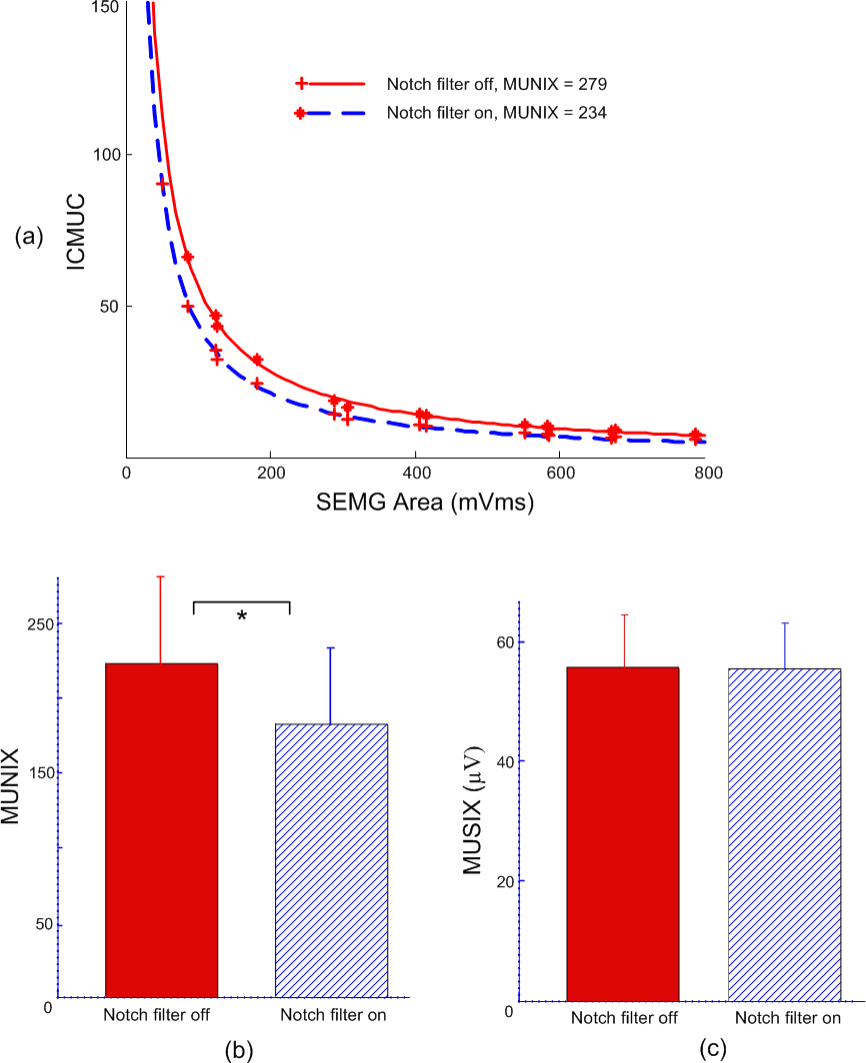
**The effects of the notch filtering function on motor unit indexes**. (a) An example of MUNIX calculation with and without notch filtering; (b) Bar plot of MUNIX values across all subjects with and without notch filtering; (c) Bar plot of MUSIX values across all subjects with and without notch filtering. "*" indicates significant difference (p < 0.001).

Maximum M wave recordings, in combination with voluntary surface EMG at different muscle contraction levels, were used to derive the MUNIX measurements. Figure 2a demonstrates an example of the MUNIX calculation, where the maximum M wave was recorded with presence and absence of the notch filtering function (10.0 mV and 13.7 mV, respectively). Analysis of SIP measurements from minimal to maximum voluntary muscle contraction in different steps (the individual data points in Figure 2a) shows an excellent fit with the mathematical model used to calculate the MUNIX (lines representing Equation 2). This subject showed a MUNIX value of 234 for the notch filtering on, which was lower than the MUNIX value of 279 for the notching filtering off.

Across all subjects (Figure 2b), the MUNIX value was 182 ± 51 (range: 67-243) for notch filtering on and 222 ± 58 (range: 91-300) for notch filtering off (p < 0.001). MUSIX values of FDI muscles were obtained from maximum M wave and MUNIX calculation according to Equation 4.

Across all subjects (Figure 2c), the MUSIX value was 55.7 ± 8.6 μV (range: 43.2-68.6 μV) for notch filtering on and 55.8 ± 7.7 μV (range: 44.2-67.6 μV) for notch filtering off (p > 0.4).

### Results from stroke subjects

Recordings of maximum M waves and voluntary surface EMG signals at different levels of contraction were also obtained from paretic and contralateral FDI muscles of all our stroke subjects, with and without the notch filter implemented.

**Figure 3:**
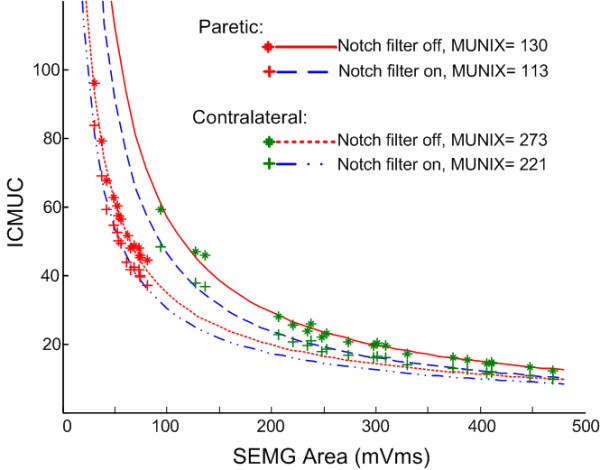
**A comparison between the MUNIX calculation from paretic and contralateral FDI muscles of the same stroke subject with and without notch filtering**.

Figure 3 demonstrates a comparison of the MUNIX calculation from paretic and contralateral muscles of one stroke subject, with notch filtering function on and off. For this stroke subject, the maximum M wave was 7.4 mV (notch filter on) and 8.9 mV (notch filter off) for the paretic muscle, compared with 12.3 mV (notch filter on) and 15.2 mV (notch filter off) for the contralateral muscle. It is worth noting that the maximum voluntary surface EMG level generated by the paretic muscle was also much lower than that from the contralateral muscle, as indicated by the x-axis values of the individual data points used for the curve fitting. With the measured maximum M wave and different levels of voluntary surface EMG values, this stroke subject showed a MUNIX value of 113 (notch filter on) and 130 (notch filter off) for the paretic FDI muscle, much lower than the MUNIX value of 221 (notch filter on) and 273 (notch filter off) for the contralateral muscle. In combination with the maximum M wave amplitudes, this resulted in MUSIX values of 65.5 μV (notch filter on) and 68.5 μV (notch filter off) for the paretic muscle, and 55.7 μV (notch filter on or off) for the contralateral muscle.

**Figure 4:**
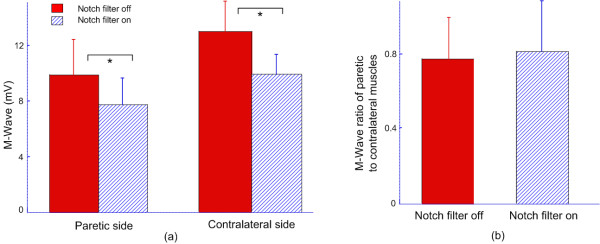
**(a) Bar plots showing the maximum M wave amplitude in presence and absence of notch filtering for paretic and contralateral muscles; (b) A comparison of the maximum M wave amplitude ratios of paretic to contralateral muscles in presence and absence of notch filtering**. "*" indicates significant difference (p < 0.001).

Figure 4 shows the effects of adding notch filtering on the maximum M wave amplitude for paretic and contralateral muscles across all stroke subjects. The maximum M wave amplitude was significantly reduced by the notch filtering for both muscles. As Figure 4a indicates, across paretic muscles, the maximum M wave amplitude was 7.8 ± 1.9 mV (range: 3.9-10.2 mV) for notch filtering on and 9.9 ± 2.5 mV (range: 5.0-13.8 mV) for notch filtering off (p < 0.001); across contralateral muscles, the maximum M wave amplitude was 9.7 ± 1.7 mV (range: 6.2-12.3 mV) for notch filtering on and 13.0 ± 2.2 mV (range: 9.8-16.2 mV) for notch filtering off (p < 0.001). Figure 4b shows the ratio of the maximum M wave amplitude in the presence and absence of the notch filtering, respectively, when the paretic muscles were compared with the contralateral ones (i.e. maximum M wave of paretic muscles divided by maximum M wave of contralateral muscles). It was observed that notch filtering does not have significant effects on the paretic-contralateral M wave ratio.

**Figure 5:**
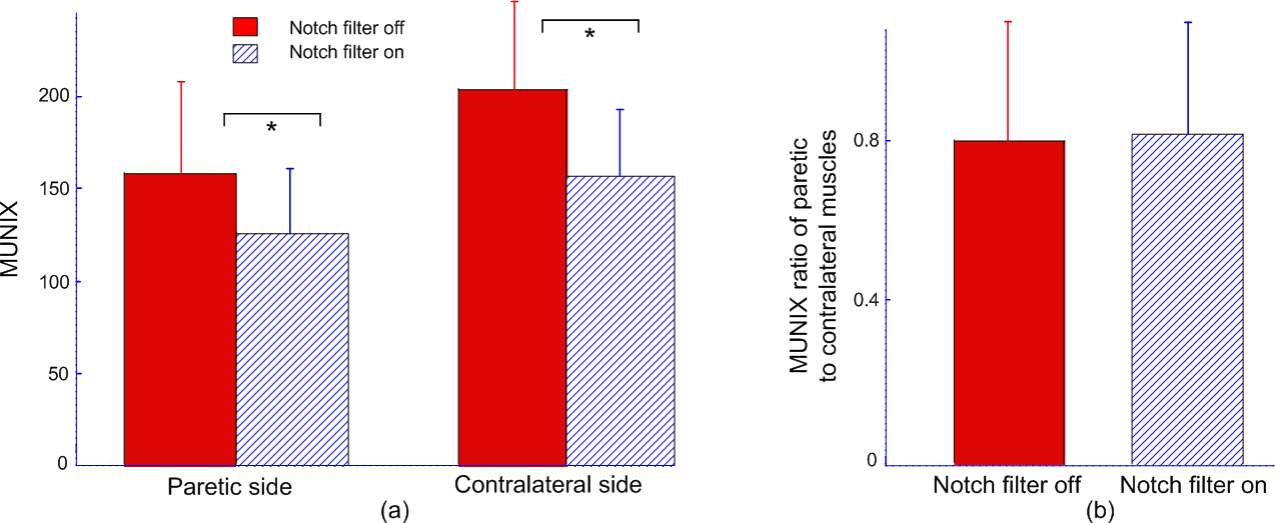
**(a) Bar plots showing the MUNIX values in presence and absence of notch filtering for paretic and contralateral muscles; (b) A comparison of the MUNIX ratios of paretic to contralateral muscles in presence and absence of notch filtering**. "*" indicates significant difference (p < 0.001).

For all the stroke subjects, exponential regression analysis in Equation 2 showed a good fitting for the relationship between SIP area and ICMUC. Figure 5 shows the effects of notch filtering on the MUNIX for paretic and contralateral muscles across all stroke subjects. Similar to findings in maximum M wave amplitude, the MUNIX was significantly decreased by the notch filtering for both muscles. Across paretic muscles, the MUNIX was 126 ± 35 (range: 56-179) for notch filtering on and 158 ± 49 (range: 74-264) for notch filtering off (p < 0.001); across contralateral muscles, the MUNIX was 158 ± 35 (range: 92-221) for notch filtering on and 204 ± 47 (range: 113-273) for notch filtering off (p < 0.001) (Figure 5a). Figure 5b shows the MUNIX ratio in the presence and absence of the notch filtering, respectively, when the paretic muscles were compared with the contralateral ones (i.e. MUNIX of paretic muscles divided by MUNIX of contralateral muscles). It was observed that notch filtering does not have significant effects on the paretic-contralateral MUNIX ratio.

**Figure 6:**
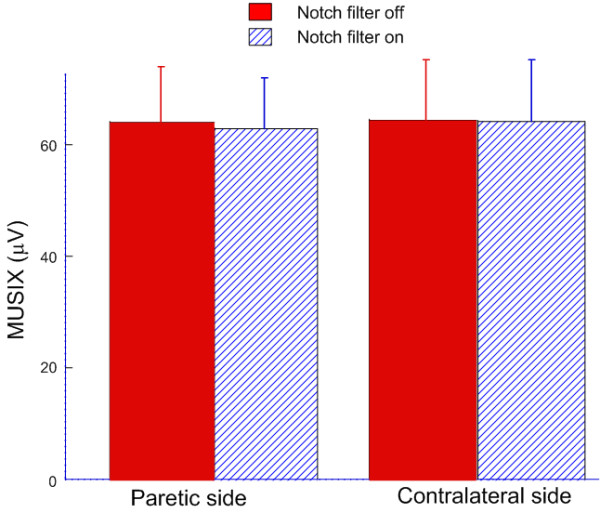
**Bar plot showing MUSIX values in presence and absence of notch filtering for paretic and contralateral muscles of stroke subjects**.

In contradistinction to our observations on maximum M wave amplitude and MUNIX measurements across all our stroke subjects, we did not observe a significant influence of notch filtering on MUSIX values. As we illustrate in Figure 6, drawn from paretic muscles of stroke subjects, the MUSIX was 62.9 ± 8.9 μV (range: 51.9- 82.7 μV) with notch filtering on and 63.9 ± 9.9 μV (range: 49.8-84.8 μV) with notch filtering off (p > 0.2). For contralateral muscles, the MUSIX was 64.3 ± 10.5 μV (range: 47.8-84.8 μV) for notch filtering on and 64.6 ± 10.3 μV (range: 49.1-84.6 μV) for notch filtering off (p > 0.8). It is worth noting that with notch filtering on or off, MUSIX values did not show significant differences between paretic and contralateral muscles (p > 0.5).

## Discussion

### Technical note

Considering that power line and harmonic noise are common during EMG recording, especially in a clinical environment with many medical or electrical supplies nearby, notch filtering is very often, if not routinely, used to suppress electromagnetic noise thus increasing the signal to noise ratio. Although earlier studies have investigated the influence of notch filtering and other electromagnetic noise suppression methods on EMG recording and other related measurements for voluntary muscle contractions [2-7], it remains unclear how such processing may alter the M wave parameters or related calculations. The present study used an experimental approach and performed a systematic examination of notch filtering effects on M wave and other relevant calculations. Our study shows that with the specific notch filter function of the EMG machine (Sierra Wave EMG system, Cadwell Lab Inc, Kennewick, WA, USA), on average the notch filtering can reduce up to more than 20% of the M wave amplitude. This could induce an average decrease in MUNIX measurement by approximately 18%. On the other hand, the notch filtering does not have significant effects on MUSIX measurement. In a previous study [22], we found relatively lower maximum M wave amplitudes for the FDI muscles when comparing with the reported values by other studies [23,24]. The findings from the present study confirm that the notch filtering processing takes a significant part in generating such a difference, although some other factors (such as subject ages) may also contribute.

It is noteworthy that the suppression of the electromagnetic noise during electrodiagnostic examination can usually be realized by online selection of the notch filtering function built into the EMG machine during the experiment while different EMG machines may have different notch filtering implementations. The findings in this report were from the analyses of the data collected using the specific EMG machine (Sierra Wave EMG system, Cadwell Lab Inc, Kennewick, WA, USA), where a 1^st ^order notch filter was implemented at the rejection frequency of 60 Hz. A different EMG machine may result in different ratios of reduction in maximum M wave amplitude and MUNIX values, in that the notch filter function may be implemented in various approaches. For example, more distortions in M wave may be imposed if the notch filter involves multiple rejecting frequencies (i.e. harmonic frequencies such as 120 Hz, 180 Hz and so on). On the other hand, the relatively significant waveform distortion shown in this study (Figure 1) may be from the low order (1^st ^order) implementation of the notch filter function. Increasing the orders of the filter may reduce the distortion imposed on the M wave.

### Implication for application of the MUNIX measurement

This study also investigated the effects of notch filtering on assessment of muscle fiber or motor unit loss in paretic muscles of stroke survivors, using maximum M wave recording and MUNIX calculation. The maximum M wave amplitude provides a reasonable approximation of the total number of muscle fibers in a muscle (or the number of motor units if the average motor unit size remained unchanged after stroke), while MUNIX measurement provides an index of the motor unit numbers in the muscle. Compared with the traditional MUNE methods that involve estimates of single motor unit potential size using either incremental nerve stimulation or spike triggered averaging techniques (both potentially laborious and time-consuming), the most advantage of the MUNIX measurement does not lie in the improved performance for adequate estimation of motor unit numbers. Instead, the most advantage of the technique is that it requires minimum amounts of electrical stimulation and is convenient and quick to implement. After its development, the technique has been successfully used to detect motoneuron loss and measure disease progression in amyotrophic lateral sclerosis and other related neuromuscular diseases [18,22,25-30]. In some patients with neurologic disorders or motoneuron diseases, the ability in activating motor unit pool may be impaired, thus constraining the voluntary EMG generation [31-33]. Considering that the MUNIX model relies on different levels of voluntary surface EMG signals, three criteria were usually imposed to accept a segment of voluntary EMG as a valid SIP epoch for MUNIX calculation, which can effectively reduce the artifacts in MUNIX estimation induced by the very low amplitude voluntary surface EMG signals [18].

As previous MUNIX studies have pointed out [19,20], the MUNIX computation is not a direct estimation of the motor unit number, and therefore, its values may not match the actual motor unit numbers estimated using other more classical MUNE methods [16,17]. When MUNIX methods are used, it should be emphasized that the objective of the study is to compare the MUNIX changes in different muscles (e.g., in neurologically intact and disease state muscles), or to compare the MUNIX changes in the same muscles in a longitudinal study (such as tracking progress of a motoneuron disease). For example, the emphasis in MUNIX examination of stroke survivors was to assess the degree of motor unit loss in the paretic muscles when compared with the contralateral ones. With the same definition for all parameters throughout the study, the absolute values of MUNIX are not important, in contrast to the changes seen from two different sides of the stroke subjects.

When examining the potential effects of notch filtering on the MUNIX measurement, our results showed that application of the notch filter function in our EMG machine (Sierra Wave EMG system, Cadwell Lab Inc, Kennewick, WA, USA) reduced the maximum M wave amplitude and the absolute values of MUNIX for all the examined muscles. On the other hand, the notch filter function on and off resulted in similar reduction ratios of both M wave and MUNIX measurements when paretic and contralateral muscles in stroke subjects were compared. This implies that notch filtering has similar effects on paretic and contralateral muscles of stroke survivors and may not alter the evaluation of the muscle fiber or motor unit loss in the paretic muscles when compared with the contralateral muscles. This also suggests that when using MUNIX, it is very important to keep the notch filtering settings the same (in the same EMG machine) throughout the study to obtain reliable physiological or diagnostic information.

### Spinal motoneuron involvement after a brain lesion

Although this study was oriented towards examining the effects of notch filtering on M wave and other related motor unit index measurements, the findings also provide evidence supporting the concept of motoneuron degeneration after a brain lesion. Previous studies have provided conflicting information as to whether muscle weakness and other functional/anatomical changes associated with stroke involve injury of spinal motoneurons. For example, using different EMG techniques (e.g., concentric needle EMG, single fiber EMG, macro-EMG and conventional surface EMG), some investigators have reported the presence of electrophysiological abnormalities as evidence of spinal motoneuron involvement, while others have not made such findings [34-36]. The abnormal EMG findings from paretic muscles of stroke survivors include fibrillation and positive sharp waves [37-40], spontaneous motor unit activities [41], increased MUAP size and complexity [39,42], increased muscle fiber density [39,43], disorganization of motor unit control properties (i.e., recruitment and firing rate) [32], and increased slope of surface EMG-force relation [44,45]. In addition, although no significant difference was found in morphometric anterior horn cell numbers of the affected and unaffected sides in stroke [46,47], variable levels of functional motor unit number reduction on the hemiparetic side was reported in stroke patients, as revealed by several MUNE methods based on incremental stimulation techniques [48-51]. Consistent to previous findings [52,53], this study shows a significant reduction in the maximum M wave amplitude in the paretic muscles compared with the contralateral or neurologically intact muscles. The MUNIX values were also found to be significantly lower in paretic muscles of stroke survivors. No significant difference in the MUSIX values was found between paretic and contralateral muscles. One potential explanation for lack of difference in MUSIX is that the paretic muscles may experience several pathological changes, for example, atrophy or denervation of muscle fibers and the reinnervtaion of muscle fibers as a compensatory process after motoneuron degeneration. Muscle fiber atrophy or dennervation may result in decreased MUSIX values while muscle fiber reinnervation may result in increased MUSIX values. Thus, the mean MUSIX values may not change dramatically when compared with the contralateral muscles.

The findings from motor unit index analysis provide further electrophysiological evidence of spinal motoneuron involvement following a stroke, suggesting that M wave and motor unit index measurements in stroke have important clinical value for the diagnosis of chronic stroke, the improvement of outcome measurements, and evaluation of the effects of medication or therapies.

## Conclusions

This study quantitatively assessed the effects of notch filtering on electrically evoked myoelectric signals and the related motor unit index measurements. The study was primarily based on an experimental comparison with the built-in notch filter function of the EMG machine (Sierra Wave EMG system, Cadwell Lab Inc, Kennewick, WA, USA) turned on and off, respectively. On average, for intact subjects, the M wave amplitude and MUNIX value of the FDI muscles were reduced by approximately 22% and 18%, respectively, with application of the notch filter function of the EMG machine. This trend held true when examining the paretic and contralateral muscles of stroke subjects. With the notch filter on vs. off, across stroke subjects, we observed a significant decrease in both maximum M wave amplitude and MUNIX values in the paretic FDI muscles, as compared with the contralateral muscles. However, similar reduction ratios were obtained for both M wave amplitude and MUNIX values. Across muscles of both intact and stroke subjects, it was observed that notch filtering does not have significant effects on MUSIX measurement. No significant difference was found in MUSIX values between the paretic and contralateral muscles of stroke subjects.

## Competing interests

The authors declare that they have no competing interests.

## Authors' contributions

XL performed human subject recruitment, experiment design, data collection and analysis, interpretation of the results, and drafting of the manuscript. WR was involved in interpretation of the results and revision of the manuscript. GL was involved in interpretation of the results. PZ oversaw the study and was involved in every stage of the study, including critical revision of the manuscript. All authors read and approved the final manuscript.
